# Phenotypic modulation of auto-reactive cells by insertion of tolerogenic molecules via MSC-derived exosomes

**Published:** 2012

**Authors:** Aram Mokarizadeh, Nowruz Delirezh, Ahhmad Morshedi, Ghasem Mosayebi, Amir-Abbas Farshid, Bahram Dalir-Naghadeh

**Affiliations:** 1 *Department of Microbiology, Faculty of Veterinary Medicine, Urmia University, Urmia, Iran; *; 2 * Department of Immunology and Microbiology, Faculty of Medicine, Arak University of Medical Sciences, Arak, Iran; *; 3 *Department of Pathobiology and Electron Microscope Center, Urmia University, Urmia, Iran; *; 4 *Department of Clinical Sciences, Faculty of Veterinary Medicine, Urmia University, Urmia, Iran.*

**Keywords:** Auto-reactive cell, EAE, MSC, Exosome, Tolerogenic molecule

## Abstract

Auto-reactive cells-mediated immune responses are responsible for the current tissue damages during autoimmunity. Accordingly, functional modulation of auto-reactive cells has been a pivotal aim in many of recent studies. In the current study, we investigated the possibility for insertion of regulatory molecules onto auto-reactive cells through exosomal nano-shuttles as a novel approach for phenotype modification of auto-reactive cells. The exosomes were isolated from supernatant of mesenchymal stem cells culture. Resultant exosomes co-cultured with lymphocytes were harvested from established EAE mice in the presence of antigenic MOG_35-55 _peptide. After 24 hr, insertion of exosomal tolerogenic molecules (PD-L1, TGF-β, galectin-1) onto auto-reactive cells were explored through flow cytometry. The potency of exosomal inserted membrane molecules to modulate phenotype of auto-reactive lymphocytes was assessed upon ELISA test for their-derived cytokines IFN-γ and IL-17. Incorporation of exosomal molecules into lymohocytes’ membrane was confirmed by flow cytometric analyses for surface levels of mentioned molecules. Additionally, the decreased secretion of IFN-γ and IL-17 were detected in exosome pre-treated lymphocytes upon stimulation with MOG peptide. Mesenchymal stem cells -derived exosomes showed to be efficient organelles for insertion of bioactive tolerogenic molecules onto auto-reactive cells and modulation of their phenotypes.

## Introduction

Exchange of membrane-fragment containing particles is described as a common mode of cell to cell communication.^1^^,^^2^ The functional consequences of such a transfer may be included in induction, amplification and/or modulation of immune responses. Moreover, induction of phenotypic modification or addition of new functional properties to recipient cells, has been described as the other possible consequences of such membrane exchanges.^3^^,^^4^

Exosomes are described as the main vesicular carriers implicated in intercellular communication. They are microvesicles released from cells through endosomal compartment and serve as shuttles for transferring of a selective pattern of surface molecules, bioactive components and genetic materials in/onto target cells.^5^^,^^6^

Mesenchymal stem cells (MSCs) are defined as multipotent non-hematopoietic progenitor cells with unique immunosuppressive capacities.^7^They have been shown to express a wide variety of regulatory molecules (PD-L1, membrane-bound TGF-β, galectins, MHC-Q2a).^7^^-^^10^ It was suggested that the-derived exosomes may harbor largely MSC-specific regulatory molecules. These exosomes transfer membrane molecules through their ability to bind target cells. However, the increasing evidence indicate that exosomal membrane integrity in recipient cells may be sufficient to favor functional and phenotype changes.^3^^,^^4^

Experimental autoimmune encephalomyelitis (EAE) is a demyelinating autoimmune disease of central nervous system mediated by myelin-specific lymphocytes specially TCD4 cells.^11^ In EAE, modulation of auto-reactive cells to attenuate their deleterious immune responses is an effective therapeutic approach. We investigated the possibility of phenotypic modulation of auto-reactive cells by inserting of exosomal tolerogenic molecules.

## Materials and Methods

**EAE induction.** Female C57BL/6 mice at 6-8 weeks old were purchased from Pasteur Institute, Tehran, Iran. All animals were housed and cared under pathogen-free conditions at the animal house of IBUU (Institute of Biotechnology of Urmia University) and treated according to the National Institute of Health Guide for Care and Use of Laboratory Animals.

EAE induction was performed according to the previously published protocol with the following modifications.^12^ Briefly, mice were immunized subcutaneously with 200 µg of MOG_35-55 _peptide (AnaSpec, Fremont, CA, USA) in 100 μL sterile PBS completely emulsified in 100 µL Complete Freund Adjuant (Sigma-Aldrich, St. Louis, MO, USA). Immunization was followed by intraperitoneal administration of 400 ng pertusis toxoid (Sigma-Aldrich, St. Louis, USA) in 400 µL sterile PBS on day O and after 48 hr.

**Isolation and proliferation of MSCs. **MSCs were isolated and expanded from flushed bone-marrows (tibias and femurs) of healthy 6-8 weeks C57/BL6 mice. After two washing by centrifugation at 1500 rpm for 10 min in PBS, harvested cells were maintained in DMEM LG medium (Invitrogen, Carlsbad, CA, USA) supplemented by 10% FBS (Invitrogen, Carlsbad, CA, USA) and antibiotics (Penicillin/ Streptomycin,100 IU per µg mL^-1^) at 37 ˚C and 5% CO_2_. After incubation for 12 hr, non-adherent cells were removed and medium was refreshed every three days. The adherent cells at confluency 70% were harvested with 0.05% trypsin solution (Sigma-Aldrich, St. Louis, MO, USA) and sub-cultured to the next passage. Mesenchymal stem cells at the third passage were used for exosome isolation.

**Flow cytometric analyses of MSCs. **Bone-marrow derived MSCs were examined for the surface mesenchymal molecules expression using flow cytometry. Briefly, 10^5^ cells were stained using the following anti-mouse monoclonal antibodies, anti-CD90 PE, anti-Sca-1 PE, anti-CD73 biotin-conjugated to streptoavidin FITC and anti-mouse CD45 FITC. Same-species and same isotype FITC or PE conjugated IgG were used as negative controls (All antibodies purchased from eBioscience, San Diego, CA, USA). Flow cytometric analyses were performed using a PAS flow cytometer (Partec GmbH, Munster, Germany). Flow Max software was used for data analysis. A total 10000 events for each samples were acquired. 

**Exosome isolation.** Exosomes were isolated from supernatant of MSC culture at the third passage according to a published protocol.^13^ Briefly, collected supernatants were serially centrifuged at 300 *g* for 10 min, 1200 *g* for 20 min and 10000 *g* for 30 min. The final supernatants were ultracentrifuged at 100000 *g* for 2 hr (Optima XL-100K Ultracentrifuge, Beckman Coulter Inc., USA). Resultant exosome pellet was washed in sterile PBS and again centrifuged at 100000 *g* for 2 hr. The final pellet was resuspended in PBS and its content protein was quantified by Bradford assay (Sigma-Aldrich, St. Louis, MO, USA).

**Electron Microscopy.** Exosomes purified by differential ultracentrifugation, were loaded on a formvar-coated grid negatively stained with 10 µL of neutral 1% aqueous phosphotungstic acid. The grids were observed by transmission electron microscope (Philips CM 100 Bio Twin; Philips, Eindhoven, The Netherlands) at 80 KV and electron micrographs were taken.

**Preparation of** **lymphocytes from spleen.** Splenocytes were prepared by pressing and mincing of EAE mice spleen in a RPMI medium (Invitrogen, Carlsbad, CA, USA) containing plate. After passing of cells through a sterile mesh to remove debris, MNCs were isolated by centrifugation on a Ficoll (Sigma-Aldrich, St. Louis, MO, USA) gradient density. 

**Phenotype analysis of exosome pre-treated lympho-cytes.** Lymphocytes (10^6 ^cells) harvested from EAE mice were cultured alone (control) or with exosomes derived from MSCs (60 μg) in the presence of MOG peptide (5 μg mL^-1^). After 24 hr, harvested lymphocytes were trypsinized to detach exosome-cell interactions and washed two times in sterile PBS. Consequently, cells were monitored for the surface levels of regulatory molecules through flow cytometry. Briefly, co-cultured cells (10^5 ^cells) were stained using the specific following antibodies, anti-PD-L1-PE (eBioscience, San Diego, CA, USA), anti-Galectin-1 followed by PE-conjugated anti-goat IgG antibody (R&D systems) and anti TGF-β followed by PE conjugated goat anti-rabbit IgG antibody (Abcam, Cambridge, MA, USA) as principle tolergenic molecules. Relevant isotype controls were used. Flow Max software was used for data analysis. A total 20000 events for each samples were acquired.

**ELISA.** Prepared splenic lymphocytes from established EAE mice were untreated (control) or pre-treated with exosomes in a RPMI (Roswell Park Memorial Institute) 1640 medium supplemented by 10% FBS. After 24 hr, cells were trypsinized to detach unincorporated exosomes and washed two times in PBS by centrifugation. Resultant lymphocytes were re-suspended in RPMI medium and cultured for another 72 hr in the presence of MOG (myelin oligodendrocyte glycoprotein) peptide. Collected supernatants were assessed for IFN-γ and IL-17 contents by ELISA kits (eBioscience, SanDiego, CA, USA).

**Statistical analysis.** Statistical analyses were performed using PASW Statistics (Version 18, SPSS Inc., Chicago, IL, USA). The data for IL-17 and IFN-γ were compared using one way analysis of variance (ANOVA) and the Bonferroni test was used for post hoc analysis to adjust for pair wise comparisons. The results are presented as mean ± standard error mean. The level of significance was set at *p *< 0.05.

## Results

**Fig. 1:**
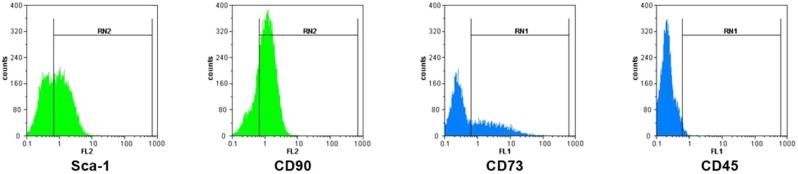
Flow cytometric characterization of mesenchymal stem cells. MSCs were positive for mesenchymal markers of CD90 (76.79), Sca-1 (55.81), CD73 (37.55) and negative for hematopoietic -lineage marker of CD45 (2.40).

**Characterization of MSCs.** A homogenous population of MSCs in spindle-shaped morphology was obtained from bone-marrow of healthy C57/BL6 mice after three passages. Flow cytometric analyses showed the expression of several stromal cell markers such as CD90, Sca-1 and CD73. MSCs did not express hematopoietic lineage marker of CD45 (Fig. 1).

**Fig. 2:**
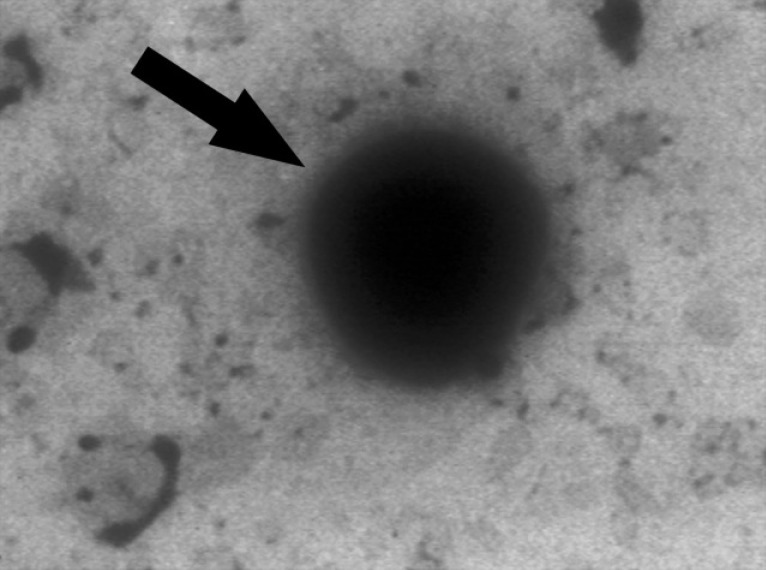
Electron microscopy analyses of exosomes. Exosome is seen as a spheroid-shaped particle in representative micrograph.

**Electron microscopy.** Exosomes were isolated from supernatant of MSC culture by differential centrifugation. Electron microscopy analyses on purified exosomes revealed presence of vesicles which mostly were smaller than 150 nm with spheroid-shaped morphology (Fig. 2).

**Fig. 3:**
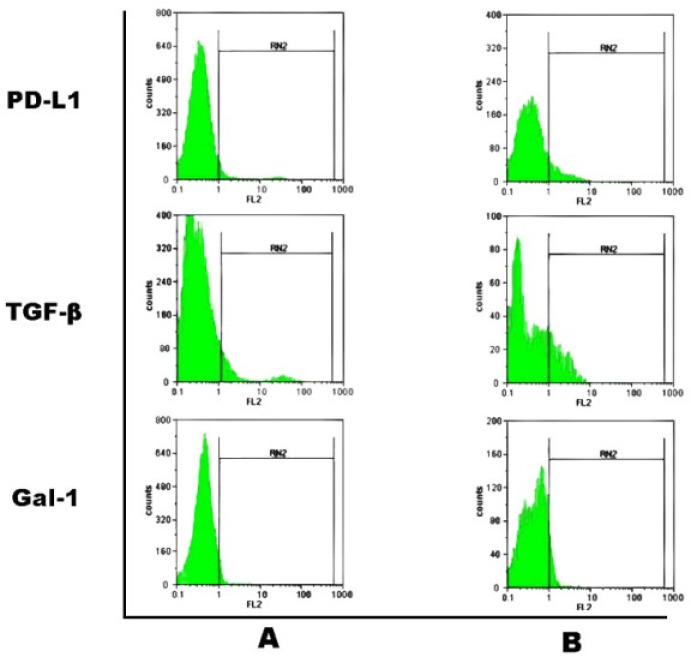
Representative histograms for flow cytometric analyses of indicated surface molecules (PD-L1, TGF-β and galectin-1) are shown. A. indicates untreated lymphocytes harvested from EAE mice; B. indicates exosome pre-treated EAE mice lymphocytes. Incorporation of exosomal components into lymphocytes’ membrane was detected as the increased expression of both MSC-specific (galectin-1) and non-specific molecules (PD-L1 and TGF-β) on lymphocytes. Mean expression of TGF-β, PD-L1and galectin-1 was found to be 20.8%, 12.5% and 11.7%, respectively. The data are presented as the mean of two independent experiments performed in duplicate.

**Incorporation of MSC-derived exosomes in MOG-activated lymphocytes.** Insertion of exosomal regulatory molecules into membrane of MOG-activated lymphocytes was detected through tracking of their expression levels on lymphocytes using flow cytometry. Results obtained from flow cytometric analyses confirmed the incorporation of exosomal membrane onto activated lymphocytes by variable increase in surface expression of PD-L1, TGF-β and galectin-1 (Fig. 3).

**Fig. 4:**
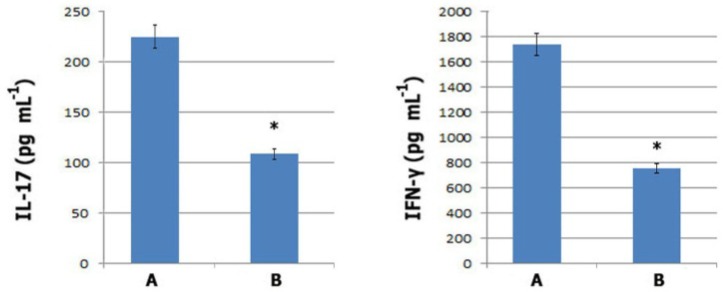
Quantitative analyses of IFN-γ and IL-17 levels by ELISA. Untreated: A. or exosome pre-treated; B. EAE mice splenic lymphocytes were trypsinized, washed and consequently cultured for 72 hr in the presence of MOG peptide. Culture supernatants were tested for mouse IFN-γ and IL-17 by ELISA. Secretion of both cytokines was significantly decreased as compared to the control untreated lymphocytes (n=4 in each group, * indicates *p *  0.001).

**ELISA.** Inflammatory cytokines of IFN-γ and IL-17 are described as the constitutional hallmarks of effector TH1 and TH17 phenotypes,^11^ Therefore, ELISA tests were performed to confirm the altered cytokine profile of exosome pre-treated lymphocytes. Results from ELISA tests showed that in exosome pre-treated lymphocytes secretion of both cytokines (IL-17 and IFN-γ) were dropped by up to 50% compared to the untreated lymphocytes (*p * 0.001) (Fig. 4). These findings confirmed the bioactivity of exosomal inserted membrane-components to modulate phenotype of auto-reactive T cells.

## Discussion

Absence or insufficient expression of regulatory molecules on immune cells is described as one of the most major causes of progressive immune responses during autoimmunity.^14^^,^^15^ Accordingly, phenotypic modification of immune cells to express regulatory molecules seems to be a potentially intriguing approach in treatment of autoimmune diseases.

In the present study, biological insertion of regulatory molecules onto auto-reactive cells through exosomal nano- shuttles was considered as a novel approach for phenotypic modification of auto-reactive cells.

Adhesion and fusion of exosomal membrane to recipient cells through lipids or ligand-receptor inter-actions were demonstrated upon several studies.^4^ Apart from the necessity of specific ligand-receptor interactions for exosome attachment and fusion, recently it has been demonstrated that exosomal surface phosphatidyl serine, on its own, has exposed exosomes to be captured by all cell types. Moreover, some receptors were expressed on the surface of activated lymphocytes and phagocytes such as TIM4 accelerated exosome capturing.^16^

Several in vitro studies demonstrated the possibility of induction, amplification and/or modulation of immune responses upon insertion of a new membrane fragment in/onto recipient immune cells.^4^^,^^5^ The functional consequences of such membrane-fragment transferring largely depend on the molecular components in transferred membrane fragment. Thus, tolerogenic exosomes mainly comprise regulatory molecules like TGF-β, PD-L1 and Fas-L.^4^

MSCs have previously been shown to express regulatory molecules of PD-L1, TGF-β and galectin-1.^7^^-^^10^ Additionally, the results obtained from flow cytometric analyses of exosome pre-treated lymphocytes; suggest MSC-derived exosomes largely harbor MSC-specific tolerogenic molecules.

PD-L1 as a negative co-stimulatory molecule, plays a key role in negative regulation of self-reactive lymphocytes and inhibition of their activation via negative signaling on PD-1 expressing lymphocytes.^17^^-^^19^ interestingly, the inserted exosomal PD-1 and PD-L1 on lymphocytes, potently promote them to activate both negative intra-cellular and intercellular signaling pathways.

Galectin-1 is described as an endogenous lectin, contributes MSCs suppressive activities. Its roles in promotion of growth arrest and increase susceptibility of activated T cells to apoptosis at both secretory and membrane-bound forms are have been demonstrated in several studies.^9^^,^^20^^,^^21^

Receptor-bound exogenous TGF-β has previously been reported to be expressed on MSCs. Its signaling whether through intrinsic ligand-receptor interaction or exogenous insertion of ligand-receptor (derived from exosomes) onto auto-reactive cells has been shown to have a powerful anti-proliferative and apoptotic effects.^22^^-^^25^

Regarding these findings, we assessed the ability of exosomal tolergenic molecules insertion onto auto-reactive lymphocytes. Results showed MSC-derived exosomes incorporated in lymphocytes’ membrane and inserted their surface regulatory molecules. However, simultaneously horizontal transferring of regulatory molecules specific mRNA is the other possible mechanism involved in the expression or the increased expression of mentioned molecules on lymphocytes.^5^^,^^6^

The functional bioactivity of exosomal inserted membrane components on auto-reactive lymphocytes was confirmed by the significant decrease in cytokine secretion of TH1 and TH17 phenotypes upon stimulation with MOG_35-55 _peptide. Even though, the internalization of inserted molecules in response to long-term different environmental stimuli is likely, this probability would be significantly reduced following administration of a large amount of exosomes due to increased scope of cell targeting and positive feed-back of targeted cells in promotion of immunosuppression.

Finally, we introduced MSC-derived exosomes as novel biosafe nano-particles for phenotypic modulation of auto-reactive cells by insertion of their regulatory molecules on cell surface.
